# Disruption of a DNA G-quadruplex causes a gain-of-function *SCL45A1* variant relevant to developmental disorders: G-quadruplex disruption unveils gain-of-function *SCL45A1* variant in disorders

**DOI:** 10.3724/abbs.2024053

**Published:** 2024-04-24

**Authors:** Yuxi Chen, Jiang Long, Sixian Wu, Yazhen Wei, Fei Yan, Qing Li, Jierui Yan, Nannan Zhang, Wenming Xu

**Affiliations:** 1 Joint Laboratory of Reproductive Medicine Gynaecology and Paediatric Diseases and Birth Defects of Ministry of Education West China Second University Hospital Sichuan University Chengdu 610041 China; 2 The Mental Health Centre and the Psychiatric Laboratory West China Hospital Sichuan University Chengdu 610041 China; 3 National Centre for Birth Defect Monitoring Key Laboratory of Birth Defects and Related Diseases of Women and Children Ministry of Education West China Second University Hospital Sichuan University Chengdu 610041 China; 4 West China School of Pharmacy Sichuan University Chengdu 610041 China

**Keywords:** *SLC45A1*, DNA G4, transcriptional enhancement, gain of function

## Abstract

*SLC45A1* encodes a glucose transporter protein highly expressed in the brain. Mutations in *SLC45A1* may lead to neurological diseases and developmental disorders, but its exact role is poorly understood. DNA G-quadruplexes (DNA G4s) are stable structures formed by four guanine bases and play a role in gene regulation and genomic stability. Changes in DNA G4s may affect brain development and function. The mechanism linking alterations in DNA G-quadruplex structures to *SLC45A1* pathogenicity remains unknown. In this study, we identify a functional DNA G-quadruplex and its key binding site on *SLC45A1* (NM_001080397.3: exon 2: c.449 G>A: p.R150K). This variant results in the upregulation of mRNA and protein expression, which may lead to intellectual developmental disorder with neuropsychiatric features. Mechanistically, the mutation is found to disrupt DNA G-quadruplex structures on *SLC45A1*, leading to transcriptional enhancement and a gain-of-function mutation, which further causes increased expression and function of the SLC45A1 protein. The identification of the functional DNA G-quadruplex and its effects on DNA G4s may provide new insights into the genetic basis of *SLC45A1* pathogenicity and highlight the importance of DNA G4s of *SLC45A1* in regulating gene expression and brain development.

## Introduction

*SLC45A1* is located on chromosome 1q42.3 and encodes a membrane protein that is predominantly expressed in melanocytes. *SLC45A1* encodes a glucose transporter protein highly expressed in the brain, which plays an important role in the uptake and utilization of glucose by the brain [1]. Mutations in *SLC45A1* may therefore lead to neurological diseases and developmental disorders [ 2, 3]. Intellectual developmental disorder with neuropsychiatric features (IDDNPF) is a term used to describe a group of conditions that involve intellectual disability and neuropsychiatric symptoms, such as behavioral problems, mood disorders, and psychosis [ 4‒ 6]. An intellectual developmental disorder with neuropsychiatric features is a moderate intellectual disability disorder caused by autosomal recessive inheritance. Patients with this disorder typically exhibit mild seizures and neuropsychiatric abnormalities, including anxiety, obsessive-compulsive behavior, and autistic features. Mild facial dysmorphic features may also be present [ 5, 7]. These symptoms can be challenging to manage and have a significant impact on the individual’s quality of life, as well as the lives of their family members and caregivers. IDDNPF can be caused by a variety of factors, including genetic mutations, environmental factors, and brain abnormalities. Although the exact causes of IDDNPF are still being studied, there is growing evidence to suggest that genetic factors play a significant role in the development of this condition [ 5, 6]. One candidate gene that may be involved in the development of IDDNPF is *SLC45A1* [ 5, 6]. The precise mechanisms underlying the role of *SLC45A1* in these processes remain poorly understood. Therefore, further investigation of *SLC45A1* is necessary to better understand the exact mechanism by which mutations in the *SLC45A1* gene may contribute to the development of IDDNPF.

DNA G-quadruplexes (DNA G4s) are regions of DNA that are formed when four guanine bases come together to form a stable structure [ 8‒ 10]. These structures are found in the telomeres of chromosomes, as well as in other regions of the genome, and they play important roles in regulating gene expression and maintaining genomic stability [ 11‒ 13]. It is thought that changes in the stability or formation of these structures may disrupt the regulation of gene expression, leading to changes in brain development and function [ 14‒ 16]. Further research into the genetics and epigenetics of IDDNPF may help to identify new targets for treatment and intervention, and ultimately improve outcomes for individuals with this condition. However, the exact mechanism by which alterations in DNA G4 structures affect target gene expression, which contributes to the development of IDDNPF, has not been reported.

This study identified the potential DNA G4s of *SLC45A1*. By evaluating the G-quadruplex formation score and conducting gene conservation analysis, we identified the crucial site responsible for this process, c.449G>A. Bioinformatics analysis predicted the pathogenicity of this variant, and its enhanced effect on the expression of *SLC45A1* was examined in HEK293T cells. Notably, this mutation was found to disrupt DNA G4 structures on *SLC45A1*, leading to transcriptional enhancement and a gain-of-function. These findings provide new insights into the genetic basis of IDDNPF and highlight the importance of DNA G4 structures of *SLC45A1* in regulating gene expression and altering brain development in a typical clinical case of IDDNPF.

## Materials and Methods

### Circular dichroism (CD) spectrum

Nucleic acid sequence samples (5 μM) were dissolved in 10 mM Tris-HCl (pH 7.5) containing 150 mM KCl. Secondary structure formation was induced by heating the nucleic acid sequence at 95°C for 5 min and gradually cooling down to 4°C. CD melting was measured with a Chirascan-Plus CD Spectrometer (Applied Photophysics, Leatherhead, UK).

### Fluorescence emission spectrum measurement

A total of 2 μM nucleic acid sequence samples were folded in 150 mM KCl following a previously described procedure [17]. Subsequently, 2 μM NMM (NMM580; Frontier Scientific, Newark, USA) or ThT (HY-D0218; MedChemExpress, Monmouth Junction, USA) was added. The excitation wavelengths were set at 393 nm for NMM and 425 nm for ThT. Emission spectra were collected from 500 nm to 700 nm for NMM and 450–700 nm for ThT. Fluorescence spectroscopy was conducted using a Hybrid Multi-Mode Reader (BioTek, Winooski, USA) with a reaction volume of 100 μL.

### FRET spectrum and melting temperature measurement

Dual-labeled nucleic acid sequence samples with 5-FAM and 3-TAMRA at a final concentration of 200 nM were folded in 150 mM KCl or LiCl following the aforementioned protocol [17]. In FRET melting experiments, fluorescence readings with excitation at 483 nm and detection at 578 nm were performed across a temperature range of 25°C to 95°C, with a temperature gradient of 0.02°C per second. The fluorescence melting curves were determined using a QuantStudio (TM) 7 Flex system (Thermo Fisher Scientific, Waltham, USA).

### Plasmid construction and cell transfection

The full-length cDNA of *SLC45A1* was synthesized and cloned into pcDNA 3.1 3×Flag by WZ Biosciences, Inc (Columbia, USA). Wild-type and mutant plasmids of *SLC45A1* were constructed and transformed into *E*. *coli*. Plasmids were extracted from bacterial cultures after overnight shaking at 37°C in Luria-Bertani medium supplemented with 50 mg/mL ampicillin. HEK293T cells were obtained from the American Type Culture Collection (ATCC, Manassas, USA) and cultured in DMEM supplemented with 10% fetal bovine serum (Gibco, Carlsbad, USA) and 0.1% penicillin/streptomycin at 37°C with 5% CO _2_. SLC45A1 plasmids were transfected into HEK293T cells using jetPRIME (Polyplus, Strasbourg, France) for 36‒48 h according to the experimental scheme. Drug treatments included PDS (10 μM, 24 h; MedChemExpress), TMPyP4 (10 μM, 24 h; MedChemExpress), and actinomycin D (1 μM, 12 h; MedChemExpress).

### Quantitative RT-PCR

Total RNA was extracted from cells using TRIzol reagent (Invitrogen, Carlsbad, USA) and reverse transcribed into cDNA using a Revert Aid First-Strand cDNA Synthesis Kit (Thermo Fisher Scientific). Quantitative PCR was performed on an iCycler RT-PCR Detection System (Bio-Rad , Hercules, USA) using SYBR Premix Ex Taq II (TaKaRa, Dalian, China), and the 2 ^‒ΔΔCT^ method was used for data analysis. All assays were conducted in triplicate for each sample, with the *GAPDH* gene used as an internal control. The primers used for real-time PCR were as follows: human *GADPH* forward, 5′-ATGTTCGTCATGGGTGTGAA-3′, reverse, 5′-GTCTTCTGGGTGGCAGTGAT-3′; human *SLC45A1* forward, 5′-CTTGTCCTGGCTATAGGGGC-3′, reverse 5′-TCGGCGCTAAAGTCCATCAG-3′.

### Western blot analysis

Proteins were extracted using RIPA buffer containing a protease and phosphatase inhibitor cocktail (Roche, Basel, Switzerland), and 50 μg of denatured proteins were separated on 10% SDS-polyacrylamide gels and transferred onto polyvinylidene difluoride (PVDF) membranes (Millipore, Billerica, USA). After being blocked with Tris-buffered saline/Tween-20 (TBST) containing 5% bovine serum albumin (BSA) for 1 h at room temperature, the membranes were incubated with primary antibodies, including anti-FLAG (1:1000; ZEN-BioScience, Chengdu, China), anti-SLC45A1 (1:100; Sigma-Aldrich), and anti-GAPDH (1:10000; Aksomics, Shanghai, China) at 4°C overnight. The binding of primary antibodies was detected using horseradish peroxidase-conjugated goat anti-rabbit or anti-mouse IgG (1:4000; Bio-Rad). Signal intensities were measured using enhanced chemiluminescence (ECL) reagent (Millipore, Billerica, USA).

### Glucose consumption assay

Glucose consumption was measured using a glucose assay kit (APPLYGEN, Beijing, China) according to the manufacturer’s instructions. Briefly, cells were seeded in 6-well plates and allowed to adhere overnight. The plasmids were then transfected with fresh medium, and the cells were incubated for 48 h. The supernatant was collected and centrifuged at 5180 *g* for 5 min to remove debris. A 5-μL aliquot of the supernatant was mixed with 195 μL of the glucose assay reagent and incubated at 37°C for 30 min. The absorbance was measured at 550 nm using a microplate reader (Thermo Fisher Scientific), and glucose consumption was calculated based on a standard curve generated using known concentrations of glucose. All assays were performed in triplicate for each sample.

### mRNA stability assay

To assess mRNA stability, actinomycin D (Sigma-Aldrich) was used to inhibit transcription. Briefly, cells were seeded in 6-well plates and allowed to adhere overnight. The medium was then replaced by fresh medium containing 1 μM actinomycin D, and the cells were incubated for various time periods (2, 4, 6, 8 and 10 h). The next steps are the same as those described in the Quantitative PCR section. All assays were performed in triplicate for each sample.

### Statistical analysis

Data were presented as the mean±standard deviation (SD). Statistical analysis was performed using GraphPad Prism (version 8.00; GraphPad Prism Software, La Jolla, USA). Two-tailed Student’s *t-*test was utilized to compare two groups, while one-way analysis of variance (ANOVA) followed by Dunnett’s test was used for the comparison of more than two groups. Statistical significance was indicated by *P*<0.05.

## Results

### Identification of functional DNA G4 and its key binding site on *SLC45A1*

**Figure 1:**
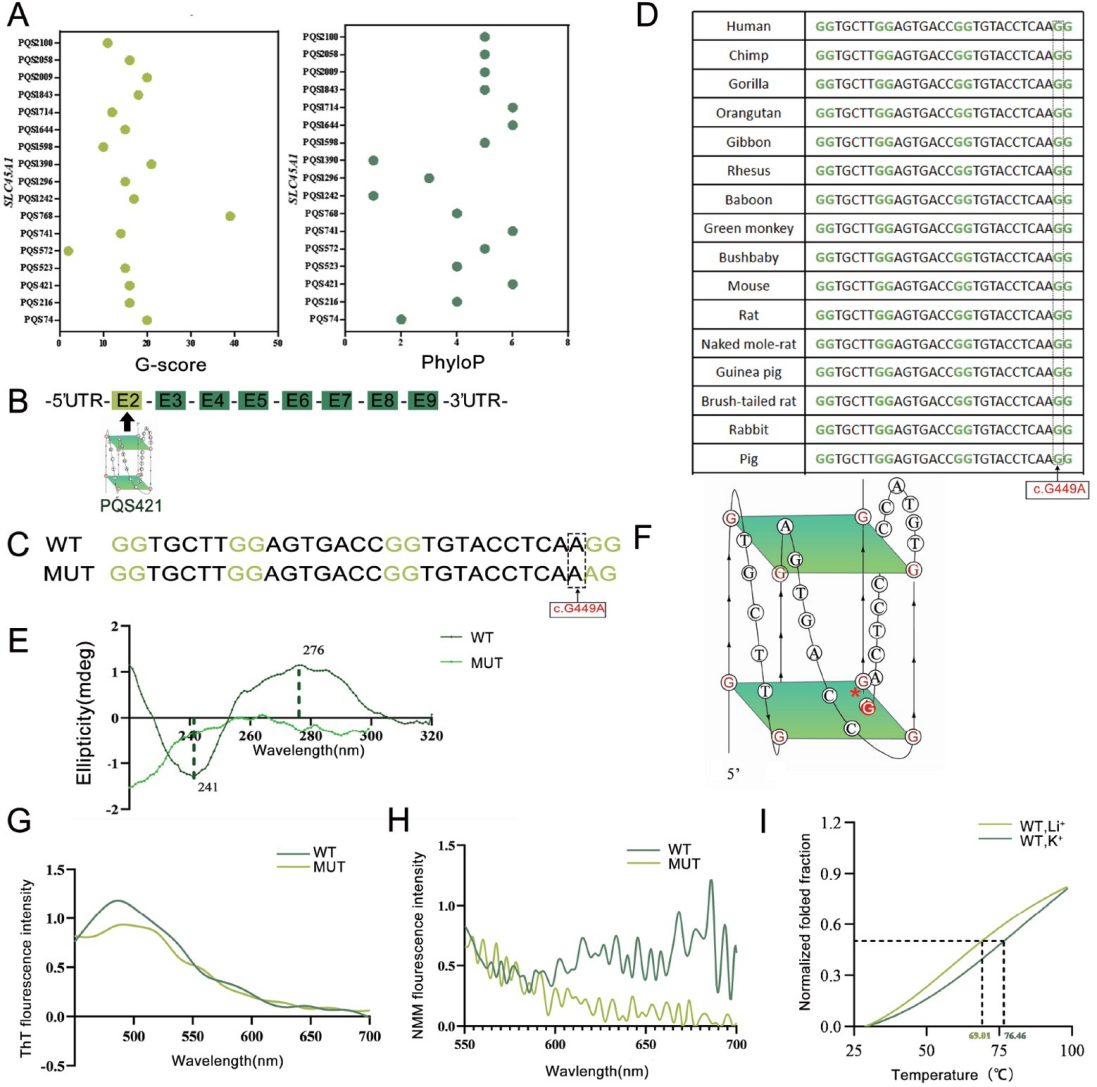
Figure 1 Characterization of DNA G4 in human *SLC45A1* (A) The potential for DNA G4 formation and sequence conservation of SLC45A1 cDNA are shown. Predicted PQSs were identified using QGRS-mapper, and the conservation of these PQSs was evaluated using the UCSC genome browser. (B) PQS421 sites in SLC45A1 gene. (C) QGRS-Mapper predicts the DNA G4 potential of the human SLC45A1 sequence and the mutant site c.449 G>A. (D) Sequence alignment reveals the conservation of the DNA G4 sequence in SLC45A1. (E) The CD spectrum demonstrates the conformation of the WT and MUT DNA G4 sequences in the presence of 150 mM KCl. (F) A schematic representation depicts the DNA G4 structure in SLC45A1, which exhibits a parallel topology and contains two G-quartets. (G) ThT and (H) NMM fluorescence emission spectra of PQ-S421-WT and PQS-421-Mut under KCl conditions. (I) FRET melting measurements of PQS-421-WT under KCl or LiCl condition.

To explore the potential involvement of DNA G4s in IDDNPF, we initially investigated putative G4-forming sequences (PQSs) in *SLC45A1* genome via in-house analysis and adopted several methods to verify the formation of DNA G4s. Seventeen PQSs were predicted in the *SLC45A1* genome by QGRS-mapper algorithm29 ( Supplementary Table S1). Sixteen regions fit the G2N1-7G2N1-7G2N1-7G2 formula, and one region fits the G3N1-7G3N1-7G3N1-7G3 formula, indicating the potential to adopt the non-canonical and metastable DG4 structures with 2 quartets or 3 quartets [ 17‒ 19]. Next, we selected the PQS421 sequence located on Exon2, which exhibits a conservative score of PhyloP>5 and a G-score >15 ( Figure 1A,B). To obtain a control sequence with a non-DNA G4 structure by disrupting the DNA G4 structure, we introduced a mutation at position c.449 G>A ( Figure 1C). The rationale for selecting the c.449 G>A mutation is that this variant was screened based on its rarity in population controls (MAF<0.001) and the functional consequence predicted by SIFT [20], PolyPhen-2 [21], and M-CAP [22]. The c.449G>A mutation was absent in EXAC Browser [23], GnomAD [24] and 1000 Genomes Project [25] ( Table 1). Functional prediction of the mutation in SIFT, PolyPhen-2, and M-CAP indicated its pathogenicity ( Table 1). Furthermore, the mutation site was highly conserved across many species ( Figure 1D) and was predicted to be highly conserved by PhyloP and PhastCons scores ( Table 1), indicating an evolutionary role of DG4 and c.449 G>A in *SLC45A1* regulation. Additionally, CD spectrum was used to examine the molar ellipticity of the putative *SLC45A1* DNA G4 sequence (23 nt) and its mutant ( Figure 1E) [ 17, 26‒ 28]. The CD spectrum of the wild-type (WT) DNA G4 sequence showed positive and negative molar ellipticity peaks at 276 nm and 241 nm, respectively, indicating a parallel DNA G4 topology [29]. However, the intensity of all the characteristic peaks decreased in MUT DNA G4 sequence ( Figure 1E). This suggests that the WT-DNA G4 sequence in *SLC45A1* could form a parallel DG4 structure ( Figure 1F) and the DNA G4 was disrupted on the c.449G>A *SLC45A1* variant. According to the fluorescence emission spectrum, PQS421 can fold into the DG4 structure, but its fluorescence intensity is weak, suggesting that it is challenging to generate more DG4 structures *in vitro* ( Figure 1G,H) [17]. FRET-melting assays were employed to assess PQS421’s thermostability. The results consistently demonstrated an increased Tm for PQS421 in the presence of KCl compared to that in the presence of LiCl ( Figure 1I) [17]. The WT-DNA G4 sequence is highly conserved ( Figure 1D), indicating an evolutionary role for DG4 in *SLC45A1* regulation. Therefore, we propose that this sequence PQS421 forms DNA G4s and c.449 G>A in *SLC45A1* DG4s is likely to play a critical role.

**Table:** **Table 1** Information of the c.449G>A in the *SLC45A1* gene

Gene	*SLC45A1*	
cDNA mutation	NM_001080397.3: c.449G>A	
Variant allele frequency	ExAC Browser	0
	GnomAD	0
	1000 Genomes Project	0
Amino acid sequence conservation	PhyloP	1.307
	PhastCons	1
Function prediction	SIFT	Deleterious
	PolyPhen-2	Probably damaging
	M-CAP	Possibly pathogenic

### The enhanced effects of disrupted DNA G4 on the expression of *SLC45A1*

**Figure 2:**
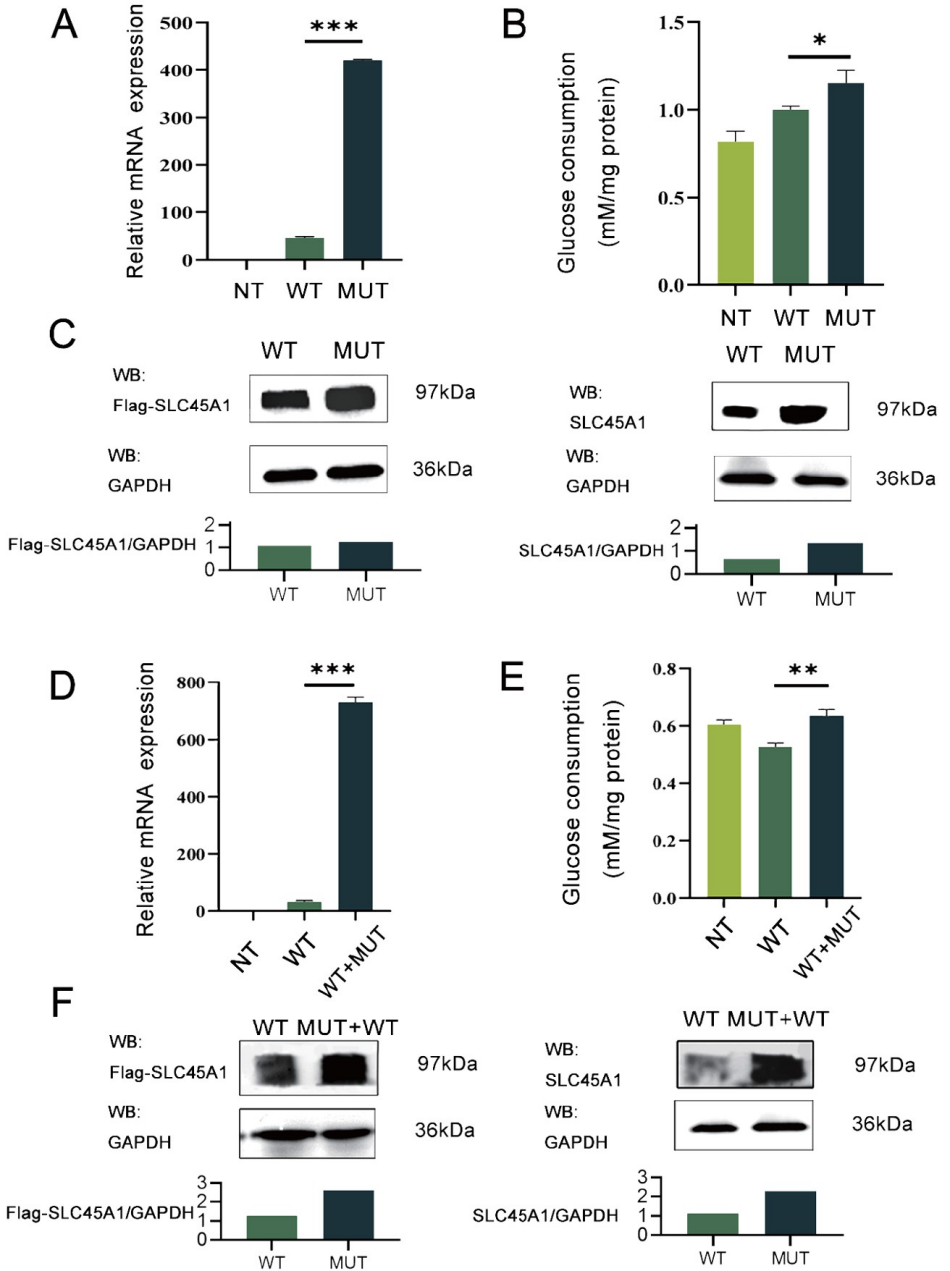
Figure 2 Molecular genetic analysis of the variants in *SLC45A1* RT-qPCR analysis demonstrated an upregulation of SLC45A1 mRNA expression in the mutation group (A) and co-transfection group (D). GAPDH was used as an internal reference gene for RT-qPCR. (B,E) Enhancement of glucose consumption in HEK-293T cells transfected and co-transfected with the SLC45A1 variant. The results are presented as the mean±SD of three individual experiments (n=3 per plasmid per experiment). Statistical significance was calculated by ANOVA in comparison with glucose uptake to WT. (C,F) SLC45A1 abundance. Lysates of the membrane fraction (50 μg protein/lane) from HEK293T cells transfected (C) and co-transfected (F) with the indicated plasmid were separated by 10% SDS-PAGE and immunoblotted with antibodies against SLC45A1 or FLAG. GAPDH was used as the loading control. ImageJ quantification of the Target protein/GAPDH ratio is shown. *P<0.05, **P<0.01, ***P<0.001.

To investigate the effect of the DNA G4 on *SLC45A1* mRNA and protein expression, we constructed wild-type (WT-Flag-pc. DNA3.1- *SLC45A1*) and mutant (MUT-Flag-pc.DNA3.1- *SLC45A1*) plasmids. Transfection of HEK293T cells with the mutant plasmid led to a significant upregulation of both SLC45A1 mRNA and protein expressions compared to transfection with the WT plasmid ( Figure 2A,C). It is noteworthy that HEK293T cells themselves scarcely express *SLC45A1*. Therefore, we simultaneously employed both FLAG antibody and SLC45A1 antibody to validate plasmid protein expression. Furthermore, the glucose transport activity of the mutant was significantly increased compared to that of the wild type ( Figure 2B).

To explore the interaction between the two strands of DNA caused by DNA G4, we co-transfected the WT and MUT plasmids into cells. The results demonstrated that the mRNA and protein expression levels of co-transfected (WT+MUT) plasmid were upregulated compared to those of wild-type plasmid ( Figure 2D,F). Additionally, the glucose transport activity of the co-transfected (WT+MUT) plasmid was also significantly increased compared to that of wild-type plasmid ( Figure 2E). These results suggested that disrupted DNA G4 caused a significant upregulation of mRNA and protein expression, leading to an increase in the function of the SLC45A1 protein.

### Disruption of *SLC45A1* DNA G4s led to the enhancement of *SLC45A1* transcriptional activity

**Figure 3:**
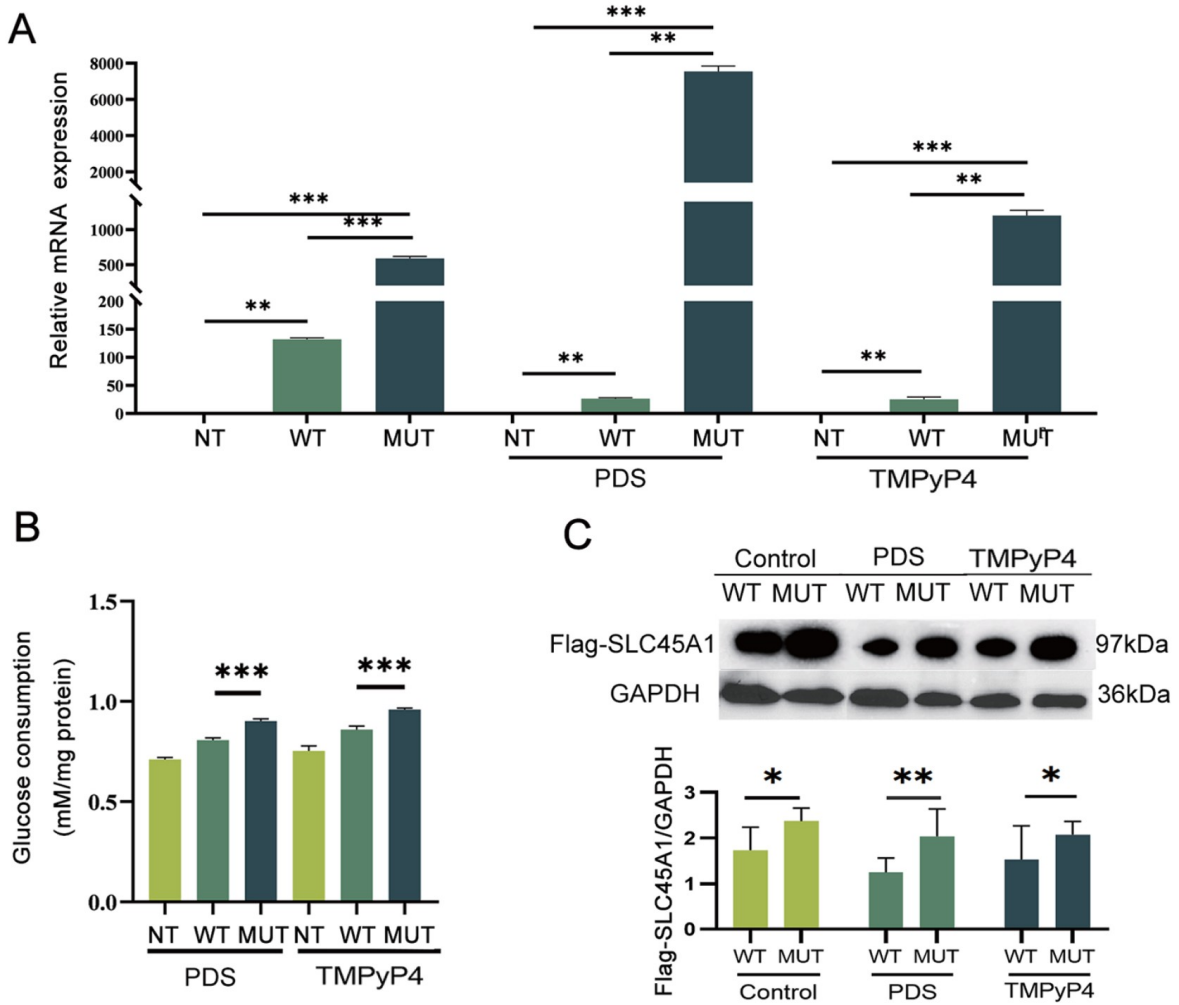
Figure 3 DG4 enhances *SLC45A1* transcription (A) Expression levels of SLC45A1 mRNA in HEK-293T cells treated with or without PDS (10 μM) or TMPyP4 (10 μM). (B) After treatment with PDS (10 μM) or TMPyP4 (10 μM), the glucose consumption of HEK-293T cells transfected with the SLC45A1 variant increased. The results are presented as the mean±SD of three individual experiments (n=3 per plasmid per experiment). Statistical significance was calculated by ANOVA in comparison with glucose uptake to WT. (C) After treatment with PDS (10 μM) or TMPyP4 (10 μM), the membrane fraction lysates (50 μg protein/lane) from HEK293T cells transfected with the respective plasmids were separated by 10% SDS-PAGE. Immunoblotting was performed using antibodies against FLAG with GAPDH as the loading control. ImageJ quantification of the Flag-SLC45A1/GAPDH ratio is shown (bottom pannel). *P<0.05, **P<0.01, ***P<0.001.

Based on these results, we investigated why the potential role of DG4 in *SLC45A1* would affect its expression and function. We transfected HEK293T cells with the wild-type plasmid and the mutant plasmid and examined the possible effect of DG4 on *SLC45A1* expression after treatment with different DG4 stabilizers. We found that all DG4 stabilizers, including PDS and TMPyP4 [ 30, 31], increased *SLC45A1* mRNA levels of the mutated *SLC45A1* compared to those of the WT ( Figure 3A). At the protein expression level, treatment of HEK293T cells with DG4 stabilizers also led to an increase in both protein expression levels and glucose transport activity of the mutated *SLC45A1* compared to those of the WT ( Figure 3B,C).

**Figure 4:**
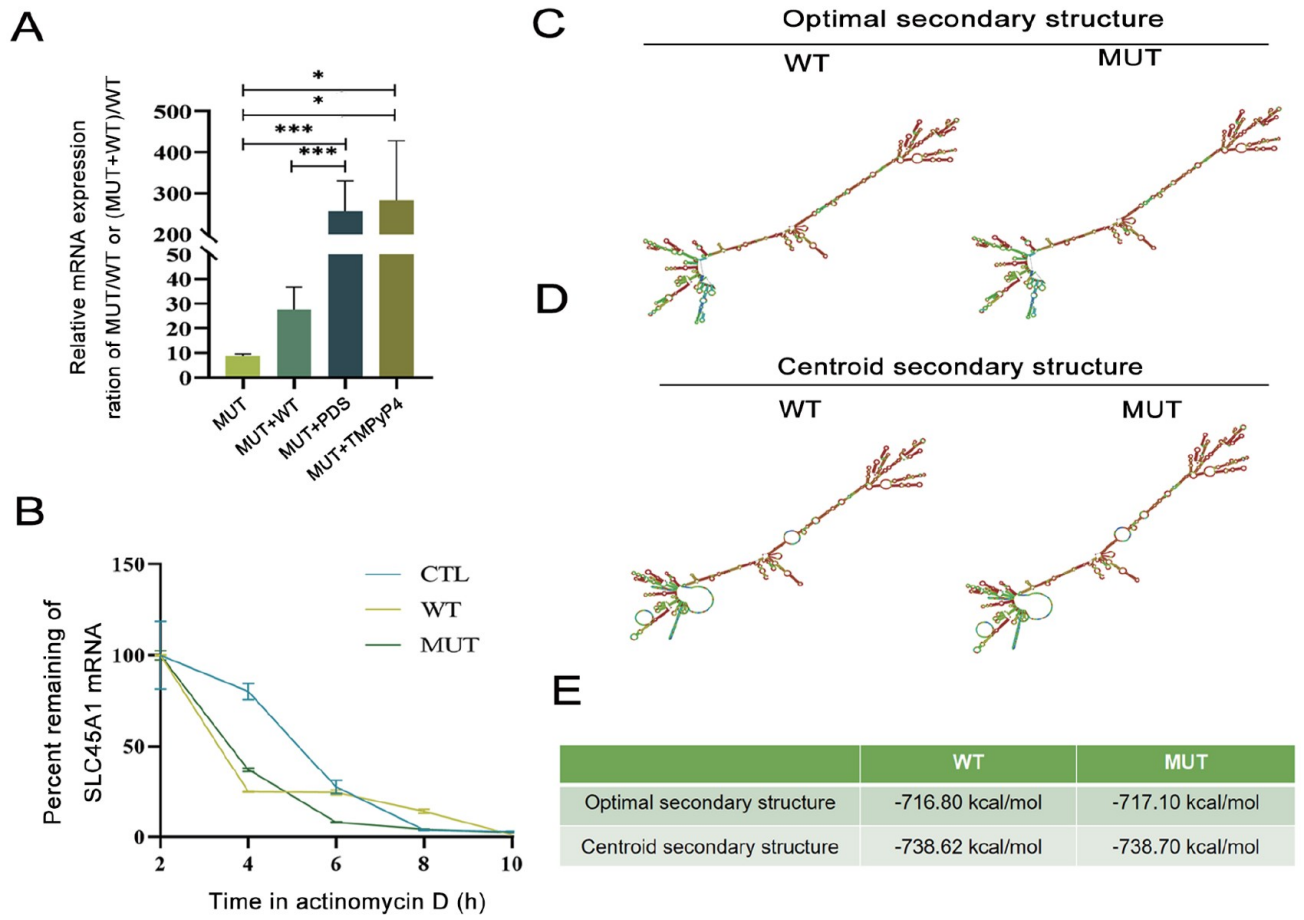
Figure 4 Expression analysis of the missense variant in SLC45A1 (A) The ratio of relative mRNA expression in HEK-293T cells transfected/co-transfected with the indicated plasmid or treated/untreated with PDS/TMPyP4. (B) RT-qPCR analysis of WT or MUT SLC45A1 mRNA expression levels in cells treated with actinomycin D to inhibit transcription after 2, 4, 6, 8 and 10 h. GAPDH was used as an internal reference gene for RT-qPCR. (C) The optimal secondary structure of the WT and MUT mRNAs determined with RNAfold. (D) The centroid secondary structure of the WT and MUT mRNAs determined with RNAfold. (E) The prediction of the minimum free energy of the mRNA secondary structure. *P<0.05, ***P<0.001.

Furthermore, after HEK293T cells were treated with DG4 stabilizer, the relative mRNA expression ratio of MUT cells and WT cells increased by more than 20 times compared with untreated cells ( *P*=0.0003 and *P*=0.0134) ( Figure 4A). In addition, the relative mRNA expression ratio of MUT and WT cells treated with DG4 stabilizers increased 10-fold ( *P*=0.0006 and *P*=0.0186) compared to the relative mRNA expression ratio of co-transfected and wild-type cells ( Figure 4A). We collected mRNA from HEK293T cells transfected with PDS-treated *SLC45A1* WT and MUT plasmids after the addition of actinomycin D [ 32, 33] to inhibit mRNA transcription. The mRNA degradation curves showed no significant difference in mRNA degradation between WT and MUT ( Figure 4B). We used RNAfold [ 34, 35] to predict the structural changes in *SLC45A1* mRNA caused by this mutation. No significant changes were observed in the mRNA structure or stability between WT and MUT ( Figure 4C‒E). These results suggest that the increased expression of *SLC45A1* mRNA after mutation is mainly due to the disruption of specific DG4 structures, leading to the enhancement of *SLC45A1* transcription rather than a significant effect on mRNA stability. These results suggest that the disruption of *SLC45A1* DNA G4s leads to the enhancement of *SLC45A1* transcriptional function, which further increases the expression and function of the SLC45A1 protein.

## Discussion

In this study, a functional DNA G4 and its key binding site of *SLC45A1* were identified. The upregulation of the expression and glucose transport activity of the variant may be responsible for its pathogenesis. Additionally, the disruption of *SLC45A1* DNA G4 was found to enhance transcription, protein expression, and function, suggesting a potential role of DNA G4 in *SLC45A1* pathogenesis and providing new insights into the regulation of brain development by *SLC45A1*.

Srour *et al*. [6] identified two homozygous missense mutations in the *SLC45A1* in two families with IDDNPF. The first mutation, c.629C-T, resulted in an A210V substitution and decreased glucose transport activity by 33%. The second mutation, c.526C-T, resulted in an R176W substitution and decreased glucose transport activity by 50%. These findings suggest that *SLC45A1* mutations may contribute to the pathogenesis of IDDNPF and provide insights into the functional effects of the identified mutations. The identification of two novel homozygous missense mutations in the *SLC45A1* expands the mutation spectrum associated with IDDNPF. These functional expression studies provide evidence for the pathogenicity of the mutations and suggest that *SLC45A1* plays a role in glucose transport activity. However, further studies are needed to elucidate the underlying mechanisms of how *SLC45A1* mutations cause disease and to explore potential therapeutic strategies for this disorder. In this study, we not only investigated a functional DG4 that enhances the *in vitro* function of *SLC45A1* and may contribute to the pathogenesis, but also explored the enhanced impact of genomic spatial structure (DNA G4) of *SLC45A1* after mutation on its transcriptional ability, which further increased its protein expression and function. Further study is required to understand the underlying mechanisms of the *SLC45A1* mutation.

At the protein expression level, treatment of HEK293T cells with DG4 stabilizers led to an increase in both protein expression level and glucose transport activity of the mutated *SLC45A1* compared to those of WT cells. However, there was no significant increase in the protein expression and function of MUT and WT cells between DG4 stabilizer-treated and DG4 stabilizer-untreated cells. These findings suggest that there may be other mechanisms affecting protein expression. The predicted destabilization energy of the mutant protein by amino acid sequence thermal stability site mutation ( http://mupro.proteomics.ics.uci.edu/) was ‒1.1887886, indicating a decrease in protein stability after the mutation. These results suggest that the decreased protein stability after *SLC45A1* mutation may contribute to the minimal difference in protein expression and function between WT and MUT cells treated with DG4 stabilizers compared to untreated cells.

In this study, we transfected a plasmid containing the expression vector for glucose transport protein but unexpectedly observed no significant alteration in the intrinsic glucose consumption of the cells. This phenomenon may stem from a combination of various factors. First, the cell’s intrinsic glucose transport system may already be saturated, as the cells themselves may possess an ample quantity of glucose transport proteins, rendering the additional expression of the protein insufficient to markedly change the rate of glucose uptake [36]. Additionally, the glucose transport rate may be constrained by other factors, such as the concentration of glucose in the culture medium. Finally, cells might regulate glucose consumption relatively stably by adjusting glucose metabolic pathways, even with increased expression of glucose transport proteins [37]. However, for the mutated plasmid, the abnormality in the protein may potentially affect signal transduction pathways associated with glucose transport. This may involve pathways such as glucose sensing and transport protein activation. Mutations could alter the normal functioning of these pathways, leading to changes in the cellular response to glucose. Moreover, the regulation of intracellular glucose metabolic pathways may also be influenced by mutations in the plasmid. Mutations could result in changes in enzyme activity or gene expression associated with glucose metabolism, thereby impacting the cell’s utilization of glucose. To gain deeper insights into this phenomenon, future research should further investigate the intracellular glucose concentration and the molecular mechanisms underlying glucose metabolic pathways. This will help unveil the intricate relationship between plasmid expression and cellular glucose metabolism, providing crucial insights for optimizing gene transfection strategies in the future.

In summary, we discovered a functional DNA G4 and its key binding site of *SLC45A1*, highlighting the importance of genetic analysis in disease ( *e.g.*, IDDNPF) diagnosis and prognosis. Additionally, our findings revealed that the DNA G4 of *SLC45A1* does not fold easily *in vitro* and serves as a crucial regulator of SLC45A1 transcription and expression, providing insight into the mechanism by which *SLC45A1* regulates brain development.

## Supporting information

23461Supplement_data

## Supplementary Data

Supplementary data is available at *Acta Biochimica et Biophysica Sinica* online.
