# Corrigendum to: Disruption of a DNA G-quadruplex causes a gain-of-function *SCL45A1* variant relevant to developmental disorders

**DOI:** 10.3724/abbs.2024184

**Published:** 2024-11-25

**Authors:** Yuxi Chen, Jiang Long, Sixian Wu, Yazhen Wei, Fei Yan, Qing Li, Jierui Yan, Nannan Zhang, Wenming Xu

Acta Biochimica et Biophysica Sinica 2024, 56(5): 709–716.

https://doi.org/10.3724/abbs.2024053

In the original version of this manuscript, an error was found in the title. Modified title is as follows. The authors apologize for this error.

**Disruption of a DNA G-quadruplex causes a gain-of-function** ***SLC45A1*** **variant relevant to developmental disorders**

