# Supplementary material for: Disruption of a DNA G-quadruplex causes a gain-of-function SCL45A1 variant relevant to developmental disorders : G-quadruplex disruption unveils gain-of-function SCL45A1 variant in disorders
Source: Acta Biochim Biophys Sin (Shanghai). 2024 Apr 24;56(5):709–16. doi: 10.3724/abbs.2024053 (PMC11177108; doi:10.3724/abbs.2024053)
Supplement: 23461Supplement_data [file 23461Supplement_data.pdf]

Supplementary Table S1. The potential DNA G4s predicted by QGRS Mapper

Position	Length	QGRS	G-Score
**74**	24	GGTCCCAGGTCACGGGCTACTCGG	20
**216**	14	GGTGGACTTCGGGG	16
**421**	30	GGTGCTTGGAGTGACCGGTGTACCTCAAGG	16
**523**	25	GGCCGGGACATTGGCATCGCCCTGG	15
**572**	30	GGGGCCTGCTGCTGACCGTGTGCGGTGTGG	2
**741**	19	GGTCGGCGGAATCCACTGGG	14
**768**	21	GGGCTTCGGGAGGGCCCTGGG	39
**1242**	29	GGACCGTGGACTTCTGGAGGGCAGAGAGG	17
**1296**	26	GGACATTCTGAGGGTGGGCTCCTTGG	15
**1390**	11	GGAGGAGGGGG	21
**1598**	20	GGTGGCTCTCATTCGAGGGG	10
**1644**	20	GGGCGAGGTGGTGTTTCAGG	15
**1714**	23	GGCGTGACCATGGGCTGCTGGGG	12
**1843**	14	GGCCTGGGGACCGG	18
**2009**	18	GGCGGGGCATGGGCGTGG	20
**2058**	30	GGCTCAGATTCTGGTCTCCCTGGTCCTGGG	16
**2100**	23	GGCCGTGGGCATGCCAACGGGG	11
